# Aurora kinase A, a synthetic lethal target for precision cancer medicine

**DOI:** 10.1038/s12276-021-00635-6

**Published:** 2021-05-28

**Authors:** Pui Kei Mou, Eun Ju Yang, Changxiang Shi, Guowen Ren, Shishi Tao, Joong Sup Shim

**Affiliations:** 1 Cancer Centre, Faculty of Health Sciences, University of Macau, Taipa, Macau SAR China; 2 MoE Frontiers Science Center for Precision Oncology, University of Macau, Taipa, Macau SAR China

**Keywords:** Targeted therapies, Drug discovery

## Abstract

Recent advances in high-throughput sequencing technologies and data science have facilitated the development of precision medicine to treat cancer patients. Synthetic lethality is one of the core methodologies employed in precision cancer medicine. Synthetic lethality describes the phenomenon of the interplay between two genes in which deficiency of a single gene does not abolish cell viability but combined deficiency of two genes leads to cell death. In cancer treatment, synthetic lethality is leveraged to exploit the dependency of cancer cells on a pathway that is essential for cell survival when a tumor suppressor is mutated. This approach enables pharmacological targeting of mutant tumor suppressors that are theoretically undruggable. Successful clinical introduction of BRCA-PARP synthetic lethality in cancer treatment led to additional discoveries of novel synthetic lethal partners of other tumor suppressors, including p53, PTEN, and RB1, using high-throughput screening. Recent work has highlighted aurora kinase A (AURKA) as a synthetic lethal partner of multiple tumor suppressors. AURKA is a serine/threonine kinase involved in a number of central biological processes, such as the G2/M transition, mitotic spindle assembly, and DNA replication. This review introduces synthetic lethal interactions between AURKA and its tumor suppressor partners and discusses the potential of AURKA inhibitors in precision cancer medicine.

## Cancer: Targeting gene combinations to kill tumors

Synthetic lethality, which arises when two genes whose functions are related in such a way that a cancer cell can survive a deficiency in one but not in both, is emerging as a viable approach to treat cancers previously regarded as not treatable by drugs. Joong Sup Shim and colleagues at the University of Macau, China, review a promising synthetic lethality strategy centered on an enzyme called AURKA that is involved in many key biological processes. If a tumor is identified as already having a deficiency in a particular gene, using a drug to inhibit the AURKA enzyme produced by a different gene can lead to cancer cell death. This is an example of personalised medicine, as it depends on identifying specific mutations in a patient’s tumor that make it vulnerable to the synthetic lethality approach.

## Introduction

Although tremendous effort has been made in cancer research over the past decades, cancer is still the leading cause of human death worldwide. Cancer arises via complex processes involving a series of genetic and epigenetic alterations. Individual cancer patients may exhibit different genetic backgrounds and carry different driver mutations, thus making it very difficult to treat every patient with a universal treatment regimen. With technological advancements in biomedical and computational sciences, complex cancer genetic landscapes have been discovered in many types of cancer, and these advancements have led to the development of precision medicines used in cancer treatment. Precision cancer medicine overcomes the influence of individual differences on the effects of drugs based on the genetic characteristics of an individual’s cancer^1^. Upon identification of driver mutations or epigenetic alterations in oncogenes or tumor suppressors in cancer, precision medicine allows clinicians to decide the most appropriate treatment options for individual cancer patients. Although this practice constitutes the future of cancer treatment, many challenges to the clinical use of precision cancer medicine need to be addressed. One of the most challenging aspects of precision cancer medicine is identified driver mutants, which not can be targeted by drugs. In particular, many tumor suppressors with loss-of-function (LOF) mutations that lead to cancer are not druggable. Treatment for cancers that develop because of oncogenic driver mutations (gene-activating or gain-of-function mutations) is relatively straightforward, as the gene product of the mutant oncogene can be pharmacologically inhibited. However, pharmacological targeting of mutant tumor suppressors (with inactivating or LOF mutations) in cancer is conceptually problematic. Synthetic lethality is one of very few possible approaches that enable pharmacological targeting of mutant tumor suppressors.

Synthetic lethality describes the phenomenon of the interplay between two or more genes in which deficiency of one gene is tolerated by a cell, which remains viable, but combined deficiency of both (or more) genes causes lethality^2^. This concept was clearly explained and its application demonstrated by W.G. Kaelin^3^. In tumors that harbor at least one gene with preexisting deficiency (e.g., LOF mutations in a tumor suppressor), pharmacological inhibition of a synthetic lethal partner of the mutant tumor suppressor can lead to selective lethality of tumor cells. However, normal cells in which the tumor suppressor gene is functional are not affected by inhibitors of the synthetic lethal partner. Since synthetic lethality provides high tumor cell selectivity, this approach has become an important part of current precision cancer medicine. This approach has been shown to be effective in cancer therapy since the successful clinical introduction of four poly (ADP ribose) polymerase (PARP) inhibitors, olaparib, rucaparib, niraparib, and talazoparib, for the treatment of breast and ovarian cancer patients with loss of the BRCA tumor suppressor^4,5^. In addition to their effects in BRCA-mutant cancers, PARP inhibitors have also been shown to induce synthetic lethality in combination with other mutant tumor suppressors, including PTEN^6^ and ARID1A^7^. PARP plays an important role in the single-stranded DNA damage repair pathway and is functionally connected with many central biological pathways, such as the DNA damage response, DNA replication, cell cycle regulation, and apoptosis. This position of PARP in the cellular network is likely to make it a common synthetic lethal partner with different tumor suppressors. Aurora kinase A (AURKA) is another possible candidate as a common synthetic lethal partner of different tumor suppressors that have recently been identified by our group and other research groups. This review introduces the cellular functions of AURKA, describes its identified interactions with synthetic lethal tumor suppressor partners, and discusses the latest developmental status of its inhibitors as potential candidates for precision cancer medicine.

### Functions of AURKA in cells and its role in cancer

An aurora kinase family is a group of serine/threonine protein kinases that regulate the cell cycle and mitotic functions and are necessary for maintaining the fidelity of genetic information. During mitosis, duplicated chromosomes are separated into the two daughter cells via the spindle. Aurora kinases participate in different steps of mitosis, and the precisely regulated spatial and temporal functions of aurora kinases are indispensable for maintaining genomic and chromosomal integrity during cell division. The aurora kinase gene was first identified in *Drosophila melanogaster* in the 1980s and was named after its defective phenotype during mitosis. Mutations in the aurora kinase gene led to impaired spindle formation, and the defective spindles formed the shape of an “*aurora*”^8^. To date, three members of the aurora kinase family have been identified in mammals: aurora A (AURKA), aurora B (AURKB), and aurora C (AURKC). The carboxyl-terminal (C-terminal) catalytic domain is highly conserved among the three aurora kinases, while the amino-terminal domain (N-terminal) varies in length and sequence among these kinases^9^. Full autoactivation of aurora kinases requires conformational changes in the C-terminal domain, which in turn requires facilitation by cofactors, such as target protein for Xenopus kinesin-like protein 2 (TPX2) for AURKA and inner centromere protein (INCENP) for AURKB/C^10–12^. After mitosis, the expression of aurora kinases needs to be maintained at a low level to enable the start of a new cell cycle. Degradation of AURKA and AURKB mainly relies on the function of E3 ubiquitin ligases. A well-known component of the degradation machinery is the anaphase-promoting complex/cyclosome (APC/C) complex, which is critical for degrading AURKA and AURKB during mitotic exit in a manner facilitated by cadherin-1 (Cdh1)^13^. Cullin 3 (Cul3)-based E3 ligases are another set of complexes required for AURKB ubiquitination^14^. The degradation pathway of AURKC remains unknown. Among the three aurora kinase family members, AURKA has been the most extensively studied, as it plays a central role in mitotic regulation and because its expression is highly associated with many types of cancer.

#### Functions of AURKA in mitosis

AURKA mainly localizes around the centrosome and the microtubule region near the centrosome. During the cell cycle, a number of AURKA proteins begin to localize near replicated centrosomes in the S phase. The majority of the AURKA population is activated in the late G2 phase, and the activity of these proteins is maintained until the end of mitosis. During mitosis, some AURKA proteins move along the mitotic spindle, while others stay near centrosomes^15^. After mitosis, most AURKA proteins undergo degradation, and only a remnant population is detected in the G1 phase. The varying distribution of AURKA proteins throughout the cell cycle reveals the potential function out of AURKA throughout this period. The functions of AURKA in mitosis mainly include centrosome maturation, mitotic entry regulation, and spindle assembly^16^.

During centriole separation, cells with defective AURKA are not able to undergo proper centriole formation (two centrioles at each pole) at both poles, while centrosome duplication and separation are not affected, suggesting that proper localization of centrioles requires AURKA function^17,18^. After centrosome duplication, centrosomes undergo a series of changes to enable their successful separation into two daughter cells after chromatin duplication. These changes include mitotic spindle formation, microtubule organization center (MTOC) formation, and pericentriolar material (PCM) recruitment. PCM proteins, including γ-tubulin, centrosomin, large tumor suppressor kinase 2 (LATS2), transforming acidic coiled-coil proteins (TACCs) and nuclear distribution element-like 1 (NDEL1), are critical for microtubule nucleation and anchoring. AURKA-defective cells exhibit impaired PCM recruitment, and later, spindle formation is obviously reduced^19^. AURKA-mediated phosphorylation of these proteins leads to their activation and/or localization to centrosomes^20,21^.

After centrosome maturation, cells can undergo mitosis. AURKA modulates mitotic entry by activating the cyclin-B/CDK1 complex, which is essential for G2/M phase entry. AURKA phosphorylates Polo-like kinase-1 (PLK1) in a manner facilitated by the cofactor Bora. Then, activated PLK1 phosphorylates CDC25 phosphatases, which are required for centrosomal activation of cyclin-B/CDK1. AURKA can also directly phosphorylate CDC25 to promote mitotic entry. On the other hand, activated PLK1 is able to degrade the CDK1 inhibitory kinase WEE1 to prevent the inactivation of cyclin-B/CDK1^22–24^. To ensure that both daughter cells inherit mitochondria from the mother cell during cell division, mitochondrial fission is necessary. During the fission process, activated cyclin-B/CDK1 phosphorylates dynamin-related protein 1 (DRP1) and targets it in mitochondria. In addition, AURKA phosphorylates the RAS family GTPase RALA and targets it to mitochondria. RALA then interacts with RALA-binding protein (RALBP1) and forms a complex with DRP1 to regulate mitochondrial fission, which is required before a cell enters the M phase^25^. AURKA can also promote M phase entry by directly phosphorylating BRCA1 at S308. This phosphorylation has been found to be reduced in cells with DNA damage-induced BRCA1-mediated G2/M arrest^26^.

During mitosis, the duplicated centrosomes separately move to the two sides of a cell and form bipolar mitotic spindles. The interaction of AURKA with TPX2 plays an important role in spindle formation. During spindle formation, activated TPX2 binds to AURKA and promotes its localization to microtubules near the centrosome and spindle pole bodies^27^. A study discovered that a large multiprotein complex that consists of AURKA, TPX2, EG5, the microtubule-stabilizing protein XMAP125/chTOG, and the microtubule-binding protein HURP is required for this spindle assembly^28^. XMAP125/chTOG is essential for the extension of microtubules from centrosomes because it stabilizes the minus end of the microtubules. However, the activity of mitotic centromere-associated kinesin (MCAK), which is critical for microtubule destabilization, is stronger than that of XMAP125 when XMAP125 functions individually. TACC3 forms a complex with XMAP125 and facilitates its ability to overcome MCAK-mediated suppression of microtubule extension. Since the localization of TACC3 to centrosomes requires its phosphorylation by AURKA, AURKA ultimately modulates the extension of microtubules from centrosomes^29,30^. During chromatin separation, kinetochore microtubules attach to the kinetochore and participate in chromatin alignment. At the same time, astral microtubules connect centrosomes to the cell cortex to anchor the centrosome, support spindle bipolarity, and ultimately promote symmetric division of duplicated chromatin. AURKA has been reported to be associated with astral microtubules^18^.

**Fig. 1:**
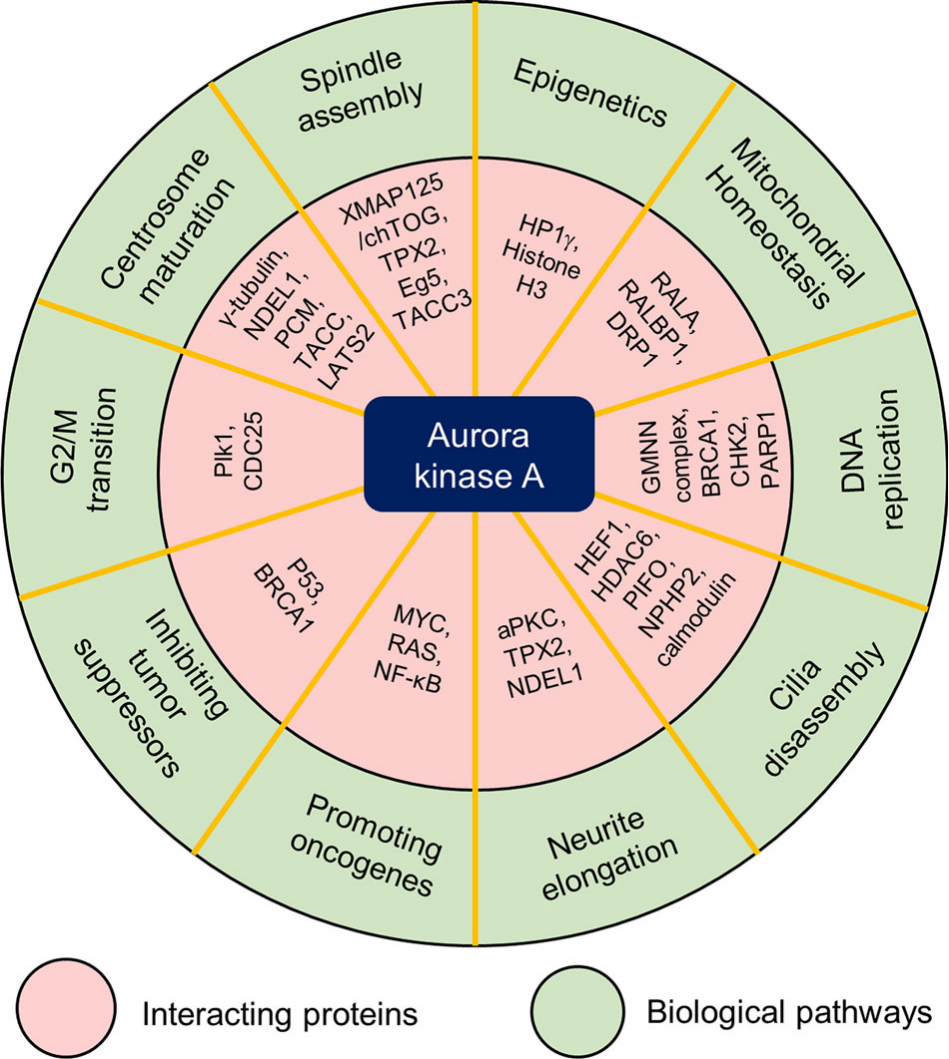
Functional diversity of aurora kinase A (AURKA). AURKA is involved in a variety of biological pathways (green circled area), including cell cycle progression, mitosis, epigenetics, mitochondrial homeostasis, DNA replication, cilia disassembly, neurite elongation, and regulation of tumor suppressors and oncogenes, through interactions or kinase-substrate relationships with its partnering proteins (pink circled area).

During the extensive study of the mitotic role of AURKA, the function of AURKA in nonmitotic cellular events has been gradually discovered. Due to its nature as a serine/threonine kinase family protein, AURKA has been found to have a variety of substrate proteins and interacting partners, including the tumor suppressor p53; epigenetic regulators, such as heterochromatin protein 1γ (HP1γ) and histone H3; atypical protein kinase C (aPKC), which is involved in neurite elongation; HDAC6, during cilia disassembly; and BRCA1, during DNA replication (summarized in Fig. 1). The functional diversity of AURKA makes it an important drug target in cancer and other pathological conditions.

#### Functions of AURKA in cancer

According to The Cancer Genome Atlas (TCGA) database, 87.9% (29 of 33) of cancer types, such as malignancies of the breast, uterus, ovaries, testes, kidneys, bladder, esophagus, colorectum, stomach, liver, lung, skin, brain, pancreas and thyroid, show high levels of AURKA expression (log2 [transcripts per million (TPM) + 1] values ≥2). Moreover, in 33 cancer types, AURKA levels were found to be elevated in almost all tumor tissues compared to the adjacent normal tissues; the exceptions were thymoma, pheochromocytoma/paraganglioma (PCPG), pancreatic adenocarcinoma (PAAD), and skin cutaneous melanoma (SKCM)^31^. Individual studies have also shown amplification or overexpression of AURKA in a number of solid and hematological cancers, including ovarian cancer^32^, breast cancer^33^, colorectal cancer^34^, esophageal squamous cell carcinoma^35^, non-small cell lung cancer^36^, bladder cancer^37^, melanoma^38^, pancreatic cancer^39^, and leukemia^40^. A high level of AURKA expression has been associated with clinical aggressiveness, poor prognosis and survival, and therapeutic resistance^36,41–45^. Hence, the role of AURKA in cancer development and the therapeutic response deserves more attention and suggests the great potential of AURKA as a therapeutic target in cancer.

A high level of AURKA expression can promote tumorigenesis by enhancing proliferation and inducing genomic and chromosomal instabilities, an antiapoptotic effect, and epithelial-mesenchymal transition (EMT). AURKA overexpression can promote cell cycle progression even when DNA is damaged or chromosomal segregation is abnormal, resulting in genomic and chromosomal instabilities, which are hallmarks of malignant tumors. For example, a high level of AURKA expression can significantly enhance CDK1 dephosphorylation, which promotes G2/M transition, via the p53, PLK1, and CDC25 pathways^23,24,46,47^. Hyperactivation of CDK1 can, in turn, inactivate DNA damage checkpoint activity and prevent apoptosis. Moreover, a high level of AURKA expression overrides spindle checkpoint assembly (SAC) activation induced by abnormal spindle formation, causing cellular aneuploidy^48^. This uncontrolled cell cycle progression leads to the accumulation of abnormal and/or defective cells in the body, which eventually contributes to malignant transformation.

AURKA levels can be positively or negatively regulated by upstream factors. Activation of positive regulators of AURKA or elimination of negative regulators of AURKA is also involved in AURKA-mediated tumor development. AURKA can be positively regulated at the transcriptional, translational, and posttranslational levels. Transcription factors, including FOXM1 and E4TF1, the chromatin-remodeling protein ARID3A, and the splicing factor PUF60, have been reported to increase AURKA transcription, whereas the mRNA-binding protein HnRNP Q1 has been reported to enhance translation efficiency by interacting with the AURKA 5’UTR^49^. The microtubule-binding protein TPX2, the translational regulator PUM2, the scaffolding protein NEDD9, and the LIM domain protein Ajuba are positive regulators of AURKA activity. They facilitate autophosphorylation of AURKA and therefore promote its kinase activity. In addition, TPX2, PUM2, and NEDD9 can protect the AURKA protein from degradation and increase its stability^50–52^. On the other hand, AURKA can also be negatively regulated at the transcriptional, posttranscriptional, and posttranslational levels. The chromatin-remodeling protein INI/hSNF5 and transcription factor SIX3 can suppress AURKA transcription by directly occupying the AURKA promoter region^53,54^. On the other hand, the ribonuclease MCPIPI can cleave AURKA mRNA at the 3’ untranslated region (UTR), resulting in destabilization of AURKA mRNA^55^. GSK-3β, PTTG1, and the protein phosphatase PP1 have been reported to inhibit AURKA activity via inhibitory phosphorylation or dephosphorylation^56–58^. Finally, IKK2, AURKAIP1, CDH1, and SMAD4 can directly interact with AURKA and trigger the degradation pathway^59–62^. In general, all these negative regulators of AURKA function as tumor suppressors, and their loss or inactivation may play an important role in AURKA-mediated cancer development^31^.

### Development of AURKA inhibitors

To target the oncogenic functions of AURKA in cancer, scientists have made great efforts to develop small molecule inhibitors of AURKA. The first generation of AURKA inhibitors is ATP-competitive catalytic inhibitors. These inhibitors mimic ATP and bind to the ATP-binding pocket. Most AURKA inhibitors currently under clinical investigation are this type of ATP-competitive inhibitor. Another type of AURKA inhibitor binds to an allosteric site, not the ATP-binding pocket, in AURKA and inhibits either its kinase activity or protein-protein interactions^63^. This is a relatively new type of AURKA inhibitor; thus, only experimental inhibitors are currently available.

To date, dozens of small molecule AURKA-specific inhibitors or pan-aurora kinase inhibitors have been developed, and 11 of these inhibitors have been evaluated in at least one complete clinical trial for patients with cancer (Table 1). Among the eleven drugs that are under clinical investigation, alisertib (MLN8237), ENMD-2076, and VX-689 (MK-5108) are specific AURKA inhibitors that have 200-fold, 25-fold, and 220-fold selectivity for AURKA over AURKB, respectively. Alisertib is the most extensively studied AURKA inhibitor in a wide range of tumor types, including lymphoma, small cell lung cancer, ovarian cancer, leukemia, gliomas, and myeloma. The results of phase 1 and 2 trials indicated that alisertib has an acceptable toxicity profile in humans. Common hematologic toxicities include neutropenia, anemia, leukopenia, and thrombocytopenia^64^. Alisertib has shown dose-limiting CNS toxicities at doses higher than 60 mg, but these CNS toxicities were found to be mitigated by dividing a single dose into two doses delivered twice daily^64^. Earlier clinical studies of alisertib were conducted with respect to hematologic malignancies. A multicenter phase 2 clinical trial of alisertib showed good antitumor activity in relapsed and refractory peripheral T cell lymphoma (R/R-PTCL)^65^. Alisertib-treated patients with R/R-PTCL who were heavily pretreated with other chemo- or targeted therapies exhibited an overall response rate of 30% (7% complete response and 23% partial response). These promising antitumor effects of alisertib in hematologic tumors led to an international phase 3 clinical trial for the same type of cancer. The phase 3 trial results indicated that while alisertib showed antitumor activity in R/R-PTCL, it showed no obvious advantage over the other comparators tested (pralatrexate, romidepsin, and gemcitabine) in the treatment groups^66^. Although alisertib as a single agent failed to show better antitumor activity than other chemotherapies or targeted therapies, many recent phase 2 trials have supported the antitumor activity of alisertib in combination therapy, especially in solid tumors. In phase 2 trials with patients with relapsed or refractory small-cell lung cancer (SCLC), patients treated with alisertib in combination with paclitaxel showed significantly improved progression-free survival (PFS) and overall survival (OS)^67^. Alisertib in combination with paclitaxel was also linked to a significant improvement in the PFS of patients with breast or ovarian cancer compared with that achieved with paclitaxel alone^68^. These results suggest that alisertib has great potential as an antitumor agent in patients with solid tumors in combination with chemotherapeutic drugs.

**Table 1:** Representative aurora kinase inhibitors and their developmental status.

Drug name	Targets	Cancer types	Completed clinical trials
Alisertib (MLN8237)	AURKA	Advanced solid tumors and lymphoma	Phase I
		Recurrent high-grade gliomas	Phase I
		Hormone receptor-positive breast cancer	Phase I
		Malignant digestive system neoplasm	Phase I
		Advanced solid tumors or colorectal cancer	Phase I
		Adenocarcinoma and pancreatic neoplasms	Phase I
		Head and neck cancer	Phase I
		Refractory multiple myeloma	Phase I/II
		Relapsed aggressive B-cell lymphoma	Phase I/II
		Neuroblastoma	Phase I/II
		Bladder cancer and transitional cell carcinoma	Phase II
		Aggressive non-Hodgkin’s lymphoma	Phase II
		Ovarian carcinoma	Phase II
		Metastatic castrate-resistant prostate cancer	Phase II
		Advanced or metastatic sarcoma	Phase II
		Recurrent leiomyosarcoma of the uterus	Phase II
		Small cell lung cancer	Phase II
		Acute myeloid leukemia	Phase II
		Relapsed/refractory peripheral T cell lymphoma	Phase III
ENMD-2076	AURKA, FLT3, Src, VEGFR2, FGFR1	Multiple myeloma	Phase I
		Relapsed hematological malignancies	Phase I
		Ovarian, fallopian and peritoneal cancer	Phase II
		Triple-negative breast cancer	Phase II
		Ovarian clear cell carcinoma	Phase II
		Advanced/metastatic soft-tissue sarcomas	Phase II
		Advanced fibrolamellar carcinoma	Phase II
VX-689 (MK-5108)	AURKA	Advanced or refractory solid tumors	Phase I
AT9283	AURKA, AURKB, JAK2, JAK3, ABL(T315I)	Advanced non-Hodgkin’s lymphoma	Phase I
		Relapsed and refractory solid tumors	Phase I
		Leukemia	Phase I
		Multiple myeloma	Phase II
MK-0457 (VX-680, Tozasertib)	Pan-Aurora kinase, FLT-3, BCR-ABL	Leukemia	Phase I/II
		Chronic myeloid leukemia and Ph+ acute lymphoblastic leukemia	Phase II
PHA-739358 (Danusertib)	Pan-Aurora kinase, ABL, TrkA, RET, FGFR1	Metastatic hormone-refractory prostate cancer	Phase II
AMG900	Pan-Aurora kinase	Acute myeloid leukemia	Phase I
		Advanced solid tumors	Phase I
AS703569 (MSC1992371A, Cenisertib)	AURKA, AURKB, ABL1, AKT, STAT5, FLT3	Pancreatic cancer	Phase I
		Solid tumors	Phase I
BI-847325	AURKA, AURKC, MEK1/2	Neoplasms	Phase I
PF-03814735	AURKA, AURKC	Advanced solid tumors	Phase I
SNS-314	Pan-Aurora kinase	Advanced solid tumors	Phase I

ENMD-2076 is another AURKA selective inhibitor that is actively being studied in clinical trials^69^. Recent results from a phase 2 clinical trial of ENMD-2076 administered as a single agent in patients with solid tumors, however, have been disappointing. ENMD-2076 led to a partial response or clinical benefit in only 16.7% of patients with pretreated, advanced, metastatic triple-negative breast cancer^70^. ENMD-2076 single-agent trials also failed to meet primary endpoints in patients with ovarian clear cell carcinoma (OCCC)^71^ or advanced soft tissue sarcoma (STS)^72^. However, significantly improved PFS or prolonged stable disease was achieved in certain groups of patients, such as patients with ARID1A-deficient OCCC or PTPRB-mutated STS, suggesting that ENMD-2076 may have clinical benefit in these molecular subtypes of cancer.

Pan-aurora kinase inhibitors have multiple targets, including the AURKA, AURKB, AURKC, FLT3, BCR-ABL, JNK, JAK2, and JAK3 kinases, with higher specificity for aurora kinases. Although pan-aurora kinase inhibitors have a higher likelihood of generating more side effects than AURKA-specific inhibitors, pan inhibitors confer some protection against the development of drug resistance in cells. To date, 8 pan-aurora kinase inhibitors—tozasertib, AMG900, AS703569, BI-847325, PF-03814735, SNS-314, AT9283, and danusertib—have been evaluated for the treatment of cancer in at least one completed clinical trial (Table 1). Based on the results of phase 1 and 2 clinical trials, all these inhibitors were generally well tolerated with manageable toxicities, while their clinical efficacies as single agents varied depending on the tumor molecular subtype. For example, tozasertib, a pan-aurora kinase inhibitor inhibiting aurora kinases, FLT3 and BCR-ABL, showed greater antitumor activity in patients with BCR-ABL T351I-mutated chronic myelogenous leukemia (CML) (44% showed hematologic responses) or Philadelphia chromosome-positive (Ph+) acute lymphoblastic leukemia (ALL) (33% showed complete remission)^73^. Most other phases 1 and 2 trials of pan-aurora kinase inhibitors as single agents showed marginal or limited clinical efficacies in hematologic malignancies or solid cancers. However, these early-stage clinical data will support the future development of aurora kinase inhibitors and the selection of appropriate target patient populations^74^.

### Identification of AURKA as a synthetic lethal target

#### The SWI/SNF nucleosome remodeling complex and AURKA

The chromatin-remodeling complex modifies chromatin architecture by moving, removing, or restructuring nucleosomes. These processes require the energy generated by ATP hydrolysis and hence are called ATP-dependent chromatin-remodeling complexes. To date, four ATP-dependent chromatin remodeling complex families have been identified, including ISWI, CHD, INO80, and SWI/SNF^75^. Among the four classes of chromatin-remodeling complexes, the SWI/SNF complex is the most extensively studied. The SWI/SNF complex was first identified in yeast and named on the basis of its defective phenotype in yeast^76^. Each SWI/SNF complex comprises 12–15 subunits and is subdivided into the BAF and PBAF subgroups based on the presence of the unique subunits in each complex^77,78^. Functionally, the SWI/SNF complex can alter the distance between nucleosomes, allowing certain regions of DNA to be exposed to transcription factors and regulate gene expression^79,80^. Loss-of-function mutations of the complex have been found in approximately 20% of human cancers^81^. As a result, the SWI/SNF complex is now generally considered a tumor suppressor. Scientists have recognized the therapeutic potential of the SWI/SNF complex in cancer and have been committed to targeting the SWI/SNF complex via the concept of synthetic lethality. To date, a number of SWI/SNF complex components have been investigated for use in synthetic lethality approaches, and interestingly, at least three components have been found to have synthetic lethal interactions with AURKA: INI/hSNF5, SMARCA4/BRG1, and ARID1A^53,82,83^.

#### Synthetic lethality between SNF5 (INI1) and AURKA in rhabdoid tumor

INI/hSNF5 is a scaffold protein in the SWI/SNF complex and has been implicated in the recruitment of transcription factors and chromatin-associated proteins. SNF5 was the first tumor suppressor identified in this complex. Loss of SNF5 is believed to be the major cause of rhabdoid tumor occurrence^84,85^. A rhabdoid tumor (RT) is a rare and highly aggressive cancer in children for which no standard of care has been established. RT mainly arises in the kidney and other soft tissues, such as the liver, muscles, peripheral nerves, or even in the brain. Currently, treatment includes surgical resection and traditional chemotherapy and radiotherapy. Because of the high aggressiveness and poor survival rate of RT, a new therapy for RT is actively being investigated.

Through microarray-based gene expression profiling, Lee et al. first discovered that *AURKA* and *PLK1* are mitotic genes repressed by SNF5^86^. Loss of SNF5 in RT cells derepressed AURKA, and the resulting overexpression of AURKA led to improper regulation of the mitotic spindle checkpoint, a major cause of RT tumorigenesis. Repression of AURKA by SNF5 likely occurs through the action of the SWI/SNF complex, which requires additional transcription factors that specifically bind to the AURKA promoter and recruit SWI/SNF to this promoter. Inhibition of AURKA expression with specific RNAi significantly inhibited the growth of RT cells. In non-RT cancer cells, such as cervical cancer and glioma cells, loss of SNF5 failed to increase the expression of AURKA, and inhibition of AURKA expression did not sensitize these cells, suggesting that synthetic lethality between SNF5 and AURKA is cancer type-specific. Silencing SNF5 also increased AURKA expression in normal fibroblast cells. However, AURKA inhibition in these cells did not cause synthetic lethality, implying the presence of a redundant mechanism that might compensate for the loss of AURKA in normal fibroblasts^53^. In summary, this study provided strong evidence showing that AURKA and PLK1 may be new drug targets that might induce selective toxicity in RT cells with SNF5 loss.

#### Synthetic lethality between SMARCA4 (BRG1) and AURKA in non-small cell lung cancer

SMARCA4/BRG1 is one of two mutually exclusive ATPases in the SWI/SNF complex. It hydrolyzes ATP to provide energy for complex functions and facilitates the activation or repression of target gene transcription. SMARCA4 is frequently mutated in many types of cancer, including lung cancer^87^, medulloblastoma^88^, and pancreatic cancer^89^. SMARCA4 was found to be mutated in approximately 19% of lung cancer cell lines evaluated, and most of these mutations were loss-of-function mutations^82^, indicating that SMARCA4 functions as a tumor suppressor in lung cancer.

To identify synthetic lethal targets in SMARCA4-mutant non-small cell lung cancer (NSCLC), Tagal et al. performed genome-wide siRNA library screening. They found 7 candidate genes, including *TPX2* and *RAN*, whose silencing induced selective toxicity against *SMARCA4*^*−/−*^ NSCLC. They then focused on AURKA because both TPX2 and RAN are upstream activators of AURKA and because AURKA is a druggable target in this pathway. Indeed, siRNA silencing or small-molecule inhibition of AURKA induced mitotic arrest and apoptosis in vitro and in xenograft mouse models of *SMARCA4*^*−/−*^ NSCLC^82^.

The centrosome-dependent and centrosome-independent pathways are two major pathways that regulate spindle assembly in mammalian cells. SMARCA4 is believed to be essential for the expression of key components of the centrosome-dependent pathway for spindle assembly, while it is thought to suppress the centrosome-independent pathway. AURKA and TPX2 are involved in both the centrosome-dependent and centrosome-independent spindle assembly machinery, but the microtubule-binding protein HURP is involved only with the centrosome-independent spindle assembly machinery^28^. Tagal et al. observed that SMARCA4 downregulated HURP protein expression. Furthermore, siRNA silencing of HURP in *SMARCA4*^*−/−*^ NSCLC cells induced mitotic arrest and apoptosis, while *SMARCA4* wild-type cells were less sensitive to HURP silencing. Considering these observations, Tagal et al. proposed that SMARCA4 is likely to be essential for supporting centrosome function during mitotic spindle assembly. In SMARCA4-deficient cells, impaired centrosome function drives cells to rely on the centrosome-independent pathway for spindle assembly, as this pathway, in some aspects, compensates for the loss of SMARCA4 function during mitosis. Inhibiting AURKA activity suppresses the centrosome-independent pathway for spindle assembly in SMARCA4-deficient cells, leading to mitotic catastrophe. This synthetic lethality model provided a list of therapeutic targets in the RAN-TPX2-AURKA-HURP axis in SMARCA4-deficient NSCLC. Although the mechanisms of the SMARCA4-induced reduction in the HURP protein level and the modulation of centrosome function by SMARCA4 remain elusive, this study established a plausible model showing an underlying synthetic lethal interaction between SMARCA4 and AURKA^82^.

#### Synthetic lethality between ARID1A and AURKA in colorectal and ovarian cancer

ARID1A refers to AT-rich interactive domain 1A, which is a BAF complex-specific subunit in the SWI/SNF complex. ARID1A contains a domain called the ARID domain, which can recognize AT-rich DNA sequences and is likely to facilitate binding of the SWI/SNF complex to DNA. As has been found for several other SWI/SNF complex components, loss-of-function mutations in ARID1A have been discovered in various cancers. Tumor histological studies showed that approximately 25.8% of colorectal primary tumors express an undetectable level of ARID1A and that another 50% of colorectal primary tumors express low levels of ARID1A (75.8% of colorectal tumors express a low or undetectable level of ARID1A)^90^. These data strongly suggest that ARID1A is an important tumor suppressor in colorectal cancer (CRC).

Wu et al. screened a human epigenetics compound library to identify synthetic lethal partners of ARID1A in ARID1A-isogenic CRC pairings. They identified AURKA inhibitors as synthetic lethal compounds in *ARID1A*^*−/−*^ CRC cells. Both siRNA silencing and small molecule inhibition of AURKA showed selective toxicity in *ARID1A*^*−/−*^ CRC cells. Reintroduction of wild-type ARID1A in *ARID1A*^*−/−*^ CRC cells significantly prevented the induction of synthetic lethality in the cells, demonstrating that AURKA inhibitor-induced synthetic lethality is dependent on the ARID1A status. The synthetic lethality of ARID1A and AURKA has been further demonstrated in ARID1A-deficient ovarian clear cell carcinoma (OCCC) cells and in mouse tumor xenograft models of CRC^83^. Mechanistically, Wu et al. found that ARID1A, as a transcriptional regulator, repressed AURKA expression by directly binding to its promoter and recruiting the SWI/SNF complex to the promoter region. ARID1A loss derepressed AURKA expression, and the AURKA level was elevated in *ARID1A*^*−/−*^ CRC cells. Consequently, CDC25C-CDK1 signaling downstream of AURKA is activated in *ARID1A*^*−/−*^ CRC cells. On the other hand, ARID1A is known to interact with ATR and is required for DNA damage-induced ATR-CHK1 signaling^7^. Activated ATR-CHK1 signaling inhibits CDC25C activity and hence blocks the G2/M cell cycle transition. Therefore, Wu et al. established a model in which the tumor-suppressive actions of ARID1A involve two inhibitory mechanisms of CDC25C function: (1) negative regulation of the upstream activator (AURKA-PLK1 axis) of CDC25C at the transcriptional level and (2) positive regulation of the upstream inhibitor (ATR-CHK1 axis) of CDC25C at the posttranslational level. Thus, loss of ARID1A promotes the AURKA-PLK1-mediated activation of CDC25C and impairs the ATR-CHK1-mediated suppression of CDC25C. These events lead to hyperactivation of the AURKA-CDC25C signaling axis in CRC cells, and these cells are thus addicted to this signaling for proliferation and survival. Thus, these oncogene-addicted cells can be selectively targeted by pharmacological inhibitors of this signaling axis, such as AURKA inhibitors or CDC25 inhibitors^83^.

Since AURKA has been found to have synthetic lethal interactions with the three aforementioned SWI/SNF complex components, the relationship between AURKA and the entire SWI/SNF complex is of interest. Wu et al. offered some speculations based on their findings. They showed that in addition to ARID1A, both SNF5 and SMARCA4 can bind to the promoter of AURKA and that this binding is ARID1A-dependent, suggesting that the entire SWI/SNF complex could bind to the AURKA promoter and regulate its transcription^83^. This result is in agreement with that of two other studies showing that SNF5 repressed AURKA expression in RT and that SMARCA4 mutations slightly but significantly increased AURKA expression in TCGA LUAD (lung adenocarcinoma) datasets. These results suggest that the synthetic lethality of AURKA might depend on the entire SWI/SNF complex, not on one or more individual components. However, since different cell/cancer types may express different systems for regulating AURKA transcription and the individual functions of SWI/SNF components can differ depending on the cell type, this model needs to be further investigated. In addition, because at least three SWI/SNF complex components have been repeatedly shown to have synthetic lethal interactions with AURKA, transcriptional repression of AURKA by these complex components may generate cellular oncogene addiction when SWI/SNF components are defective. Further investigation on AURKA as a drug target in cancers where other SWI/SNF components are frequently mutated is warranted.

#### Synthetic lethality between RB1 and AURKA in small cell lung cancer

*RB1* (retinoblastoma susceptibility gene 1) is a typical tumor suppressor gene whose loss-of-function mutation is observed in multiple aggressive and treatment-refractory malignancies. RB1 is critical for preventing excessive cell growth by negatively regulating cell cycle progression, especially the G1/S transition. RB1 functions by binding and inhibiting the oncogenic transcription factor E2F, which activates the transcription of target genes involved in cell cycle progression^91^. *RB1* is frequently mutated in small cell lung cancer (SCLC) cells, with a mutation frequency of more than 90%^92^. Unlike for NSCLC, no oncogene drug targets have been reported for SCLC. Therefore, mutant *RB1* has been considered a drug target for the treatment of SCLC. Here, we introduce two recent independent studies that identified AURKA as a synthetic lethal target partnered with RB1 in SCLC.

Gong et al. conducted pharmacogenomic profiling of 36 small molecule cell cycle inhibitors in more than 500 genome-characterized cancer cell lines. From this large-scale cancer cell profiling effort, they found that RB1-mutant SCLC cells are highly sensitive to AURKA inhibitors^93^. AURKA inhibitors, including MK5108, alisertib, and LY3295668, were found to induce selective toxicity toward *RB1*-mutant cancer cells by inducing apoptosis. The synthetic lethality of AURKA inhibitors was further verified by overexpression of a mutant (drug binding-defective) form of AURKA that protected cells against the cytotoxic effects of LY3295668. Oral administration of LY3295668 was well tolerated in rodents, and LY3295668 did not exhibit cytotoxic effects in human bone marrow cells; in addition, this treatment exhibited a strong synthetic lethal effect on *RB1*-mutant SCLC xenografts in mice. In a mechanistic study, Gong et al. conducted a genetic suppression screen using a genome-scale shRNA library to identify genes critical for AURKA inhibitor cytotoxicity. They identified seven genes whose silencing suppressed the synthetic lethal effect of the AURKA inhibitor. These suppressors were *BUB1B* and *BUB3*, which are mitotic checkpoint complex (MCC) genes critical for enforcing the spindle assembly checkpoint (SAC) by inhibiting APC/C (anaphase-promoting complex/cyclosome) activity. Based on a series of studies, Gong et al. proposed a model describing the underlying synthetic lethal interaction between RB1 and AURKA: (1) In *RB1* wild-type cells, RB1 strictly controls the expression of SAC genes, such as MAD2, and prevents the constitutive activation of SAC. AURKA inhibition is only toxic to cells when applied at high doses. (2) In *RB1-*mutant cells, SAC genes are upregulated, which leads to a primed (activated) state of SAC. A high level of AURKA kinase activity can override the effect of activated SAC. Thus, *RB1-*mutant cells constitutively require the function of AURKA to exit mitosis and to survive. This phenomenon creates a unique cellular dependency on AURKA when *RB1* is mutated^93^.

In a separate study, Lyu et al. screened human epigenetic compounds and RNAi libraries using *RB1* isogenic lung cancer cell pairs. On the basis of these screens, they identified AURKA as the strongest synthetic lethal partner of RB1 among the candidate hits^94^. AURKA siRNA and small molecule AURKA inhibitors, including ENMD-2076, Aurora A inhibitor I, and alisertib, induced selective toxicity in *RB1*^*−/−*^ lung cancer cells. All three AURKA inhibitors showed strong antitumor effects on *RB1*^*−/−*^ lung cancer xenografts in mice, while *RB1*^*+/+*^ tumors were much less sensitive to the inhibitors. A series of mechanistic studies revealed that *RB1*^*−/−*^ cells exhibit reduced microtubule stability compared to *RB1*^*+/+*^ cells and that AURKA inhibition further reduces microtubule stability in *RB1*^*−/−*^ cells. This feature of *RB1*^*−/−*^ cells endows these cells with hypersensitivity to agents targeting microtubule dynamics, including vinorelbine and paclitaxel. These phenotypic alterations in *RB1*^*−/−*^ cells led Lyu et al. to investigate a potential factor that regulates microtubule dynamics in *RB1*^*−/−*^ cells as a mediator of synthetic lethality. They conducted transcriptome profiling to compare *RB1*^*+/+*^ cells with *RB1*^*−/−*^ cells and identified a group of microtubule stability regulators elevated in *RB1*^*−/−*^ cells. Among these genes, stathmin (STMN1) was found to be an E2F target gene whose expression was highly increased in *RB1*^*−/−*^ cells. STMN1 is a phosphoprotein that upon its activation (dephosphorylation) binds and destabilizes microtubules. Lyu et al. found that transcription factors in the E2F family (E2F1-3) directly bind to the STMN1 promoter and activate its transcription. RB1 is a negative regulator of E2F and hence represses STMN1 expression. Indeed, a strong negative correlation was observed between the protein levels of RB1 and STMN1 in a panel of human lung cancer cell lines and a large panel of lung adenocarcinoma (LUAD) patient samples in TCGA datasets. Lyu et al. also showed that AURKA is an upstream kinase that phosphorylates and inhibits STMN1 activity and that inhibiting AURKA activity, in turn, activates STMN1 function to reduce microtubule stability. Microtubule staining and bipolar spindle formation assays further showed that AURKA inhibition promoted the destabilization of microtubules in unsynchronized cells and strongly suppressed bipolar spindle formation in mitotic cells^94^.

Considering these observations, Lyu et al. proposed a model in which (1) RB1 loss activates the E2F transcription factor family and hence upregulates STMN1 expression. (2) Elevated STMN1 levels in *RB1*^*−/−*^ cells facilitate microtubule destabilization, but AURKA mitigates this effect by suppressing STMN1 activity. This outcome reveals a dependency of *RB1*^*−/−*^ cells on AURKA activity. (3) AURKA inhibition leads to high levels of activated STMN1 in *RB1*^*−/−*^ cells, profound disruption of microtubule dynamics, and SAC hyperactivation, in turn leading to mitotic cell death. This model fills a gap in the model proposed by Gong et al. by showing an upstream mechanism of the change in microtubule dynamics and *RB1*^*−/−*^ cell dependency on AURKA. In summary, the studies from both groups suggest a new druggable target in SCLC and provide mechanistic insight into the synthetic lethal interaction between RB1 and AURKA.

### Future perspectives

**Fig. 2:**
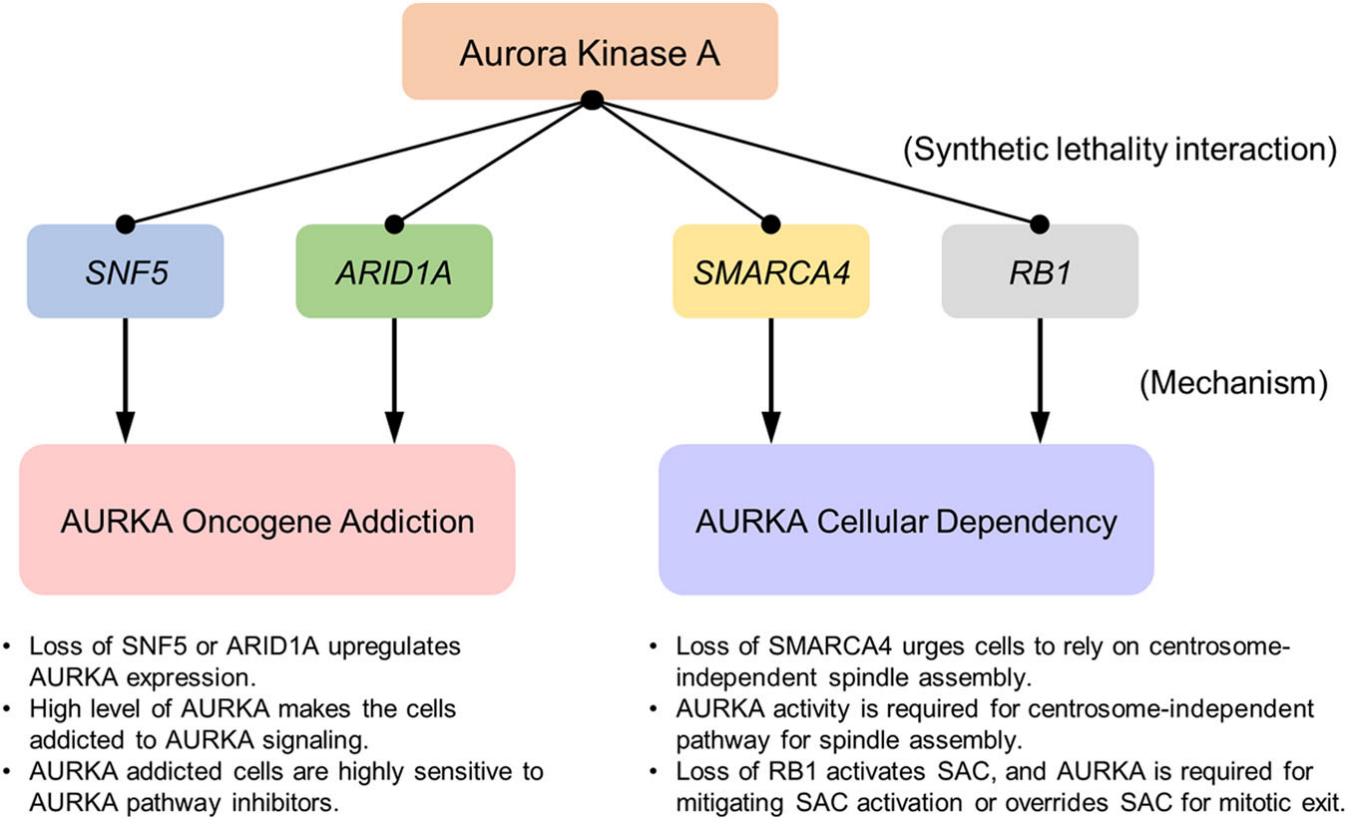
Two common mechanisms of aurora kinase A (AURKA) synthetic lethal interactions. Synthetic lethal interactions of AURKA with the SNF5 and ARID1A tumor suppressors involve SWI/SNF-mediated transcriptional repression of AURKA. Loss of SNF5 or ARID1A derepresses AURKA transcription, and the elevated AURKA level in rhabdoid tumor (RT) or colorectal cancer (CRC) cells leads to AURKA oncogene addiction. AURKA-addicted RT and CRC cells are highly sensitive to agents targeting AURKA signaling. The synthetic lethal interactions of AURKA with the SMARCA4 and RB1 tumor suppressors involve cellular AURKA dependency. SMARCA4 is likely to play an essential role in centrosome-dependent spindle assembly, while it negatively regulates the centrosome-independent pathway. AURKA is essential in both pathways for spindle assembly. Loss of SMARCA4 drives cells to rely on the centrosome-independent pathway, which creates a cellular dependency on the function of AURKA in spindle assembly. Loss of RB1 primes spindle assembly checkpoint (SAC) activation in cells via E2F-mediated upregulation of the SAC protein MAD2 and microtubule destabilizer STMN1. Active AURKA in RB1-deficient cells mitigates STMN1-induced microtubule destabilization or overrides SAC activation for cell survival.

This review introduces the synthetic lethal interactions of AURKA with four different tumor suppressors frequently mutated in cancer: AURKA-SNF5, AURKA-SMARCA4, AURKA-ARID1A, and AURKA-RB1. Although other synthetic lethal interactions of AURKA have been reported, such as AURKA-CHK1/WEE1^95,96^ and AURKA-PARP1^97,98^ interactions, we do not discuss them in this review because they more likely illustrate synergism between two compounds or show chemical synthetic lethality that does not involve tumor suppressor deficiency. The five studies discussed herein reveal two common mechanisms by which AURKA contributes to synthetic lethality with tumor suppressors. One mechanism involves AURKA overexpression after mutation of a tumor suppressor, leading to oncogene addiction in cancer cells. The other involves the generation of a unique cellular dependency on AURKA functions for their survival when a tumor suppressor is mutated (summarized in Fig. 2). Since fine-tuned regulation of AURKA activity and expression is highly important in multiple central cellular processes, such as the G2/M transition and mitosis, dysregulation of its activity or expression plays an important role in cancer development. These unique features of AURKA make it an important drug target for precision cancer medicine under the circumstances described.

Previous and ongoing clinical studies of AURKA inhibitors in cancer have not strongly focused on tumor suppressor status. However, some of the clinical studies of AURKA inhibitors have revealed that their use in particular molecular tumor subtypes leads to better clinical outcomes. Notably, the clinical trial of ENMD-2076 in patients with OCCC showed significant antitumor activity of ENMD-2076 in ARID1A-deficient OCCC subtypes^71^. This study strongly supports the feasibility of leveraging synthetic lethality between AURKA and ARID1A in clinical settings. Upon the discovery of several synthetic lethal interactions of AURKA and tumor suppressors, additional clinical studies of AURKA inhibitors that are tailored to the specific tumor suppressor status should be performed in the future. In addition, current AURKA inhibitors function mainly by targeting the catalytic pocket and preventing autophosphorylation at Thr288^99^. Accumulating evidence indicates that some of the functions of AURKA are independent of its intrinsic kinase activity^100^. Either blocking the subcellular localization of AURKA or inhibiting protein–protein interactions could sensitize drug-resistant cancer cells to kinase inhibition. Therefore, the development of chemically or mechanistically diverse AURKA inhibitors is necessary to overcome current clinical challenges and will provide diverse options for using AURKA inhibitors in precision cancer medicine.

## References

[1] Letai A (2017). Functional precision cancer medicine-moving beyond pure genomics. Nat. Med..

[2] Guarente L (1993). Synthetic enhancement in gene interaction: a genetic tool come of age. Trends Genet..

[3] Kaelin WG (2005). The concept of synthetic lethality in the context of anticancer therapy. Nat. Rev. Cancer.

[4] Farmer H (2005). Targeting the DNA repair defect in BRCA mutant cells as a therapeutic strategy. Nature.

[5] Bryant HE (2005). Specific killing of BRCA2-deficient tumours with inhibitors of poly(ADP-ribose) polymerase. Nature.

[6] Mendes-Pereira AM (2009). Synthetic lethal targeting of PTEN mutant cells with PARP inhibitors. EMBO Mol. Med..

[7] Shen J (2015). ARID1A deficiency impairs the DNA damage checkpoint and sensitizes cells to PARP inhibitors. Cancer Discov..

[8] Glover DM, Leibowitz MH, McLean DA, Parry H (1995). Mutations in aurora prevent centrosome separation leading to the formation of monopolar spindles. Cell.

[9] Cheetham GM (2002). Crystal structure of aurora-2, an oncogenic serine/threonine kinase. J. Biol. Chem..

[10] Sessa F (2005). Mechanism of Aurora B activation by INCENP and inhibition by hesperadin. Mol. Cell.

[11] Bayliss R, Sardon T, Vernos I, Conti E (2003). Structural basis of Aurora-A activation by TPX2 at the mitotic spindle. Mol. Cell.

[12] Li X (2004). Direct association with inner centromere protein (INCENP) activates the novel chromosomal passenger protein, Aurora-C. J. Biol. Chem..

[13] Stewart S, Fang G (2005). Destruction box-dependent degradation of aurora B is mediated by the anaphase-promoting complex/cyclosome and Cdh1. Cancer Res..

[14] Sumara I (2007). A Cul3-based E3 ligase removes Aurora B from mitotic chromosomes, regulating mitotic progression and completion of cytokinesis in human cells. Dev. Cell.

[15] Goldenson B, Crispino JD (2015). The aurora kinases in cell cycle and leukemia. Oncogene.

[16] Nikonova AS, Astsaturov I, Serebriiskii IG, Dunbrack RL, Golemis EA (2013). Aurora A kinase (AURKA) in normal and pathological cell division. Cell. Mol. Life Sci..

[17] Schumacher JM, Ashcroft N, Donovan PJ, Golden A (1998). A highly conserved centrosomal kinase, AIR-1, is required for accurate cell cycle progression and segregation of developmental factors in Caenorhabditis elegans embryos. Development.

[18] Giet R (2002). Drosophila Aurora A kinase is required to localize D-TACC to centrosomes and to regulate astral microtubules. J. Cell Biol..

[19] Berdnik D, Knoblich JA (2002). Drosophila Aurora-A is required for centrosome maturation and actin-dependent asymmetric protein localization during mitosis. Curr. Biol..

[20] Mori D (2007). NDEL1 phosphorylation by Aurora-A kinase is essential for centrosomal maturation, separation, and TACC3 recruitment. Mol. Cell. Biol..

[21] Hannak E, Kirkham M, Hyman AA, Oegema K (2001). Aurora-A kinase is required for centrosome maturation in Caenorhabditis elegans. J. Cell Biol..

[22] Elia AE, Cantley LC, Yaffe MB (2003). Proteomic screen finds pSer/pThr-binding domain localizing Plk1 to mitotic substrates. Science.

[23] van Vugt MA, Bras A, Medema RH (2004). Polo-like kinase-1 controls recovery from a G2 DNA damage-induced arrest in mammalian cells. Mol. Cell.

[24] Seki A, Coppinger JA, Jang CY, Yates JR, Fang G (2008). Bora and the kinase Aurora a cooperatively activate the kinase Plk1 and control mitotic entry. Science.

[25] Kashatus DF (2011). RALA and RALBP1 regulate mitochondrial fission at mitosis. Nat. Cell Biol..

[26] Brodie KM, Henderson BR (2012). Characterization of BRCA1 protein targeting, dynamics, and function at the centrosome: a role for the nuclear export signal, CRM1, and Aurora A kinase. J. Biol. Chem..

[27] Kufer TA (2002). Human TPX2 is required for targeting Aurora-A kinase to the spindle. J. Cell Biol..

[28] Koffa MD (2006). HURP is part of a Ran-dependent complex involved in spindle formation. Curr. Biol..

[29] Kinoshita K (2005). Aurora A phosphorylation of TACC3/maskin is required for centrosome-dependent microtubule assembly in mitosis. J. Cell Biol..

[30] Giet R, Uzbekov R, Cubizolles F, Le Guellec K, Prigent C (1999). The Xenopus laevis aurora-related protein kinase pEg2 associates with and phosphorylates the kinesin-related protein XlEg5. J. Biol. Chem..

[31] Du R, Huang C, Liu K, Li X, Dong Z (2021). Targeting AURKA in Cancer: molecular mechanisms and opportunities for Cancer therapy. Mol. Cancer.

[32] Gritsko TM (2003). Activation and overexpression of centrosome kinase BTAK/Aurora-A in human ovarian cancer. Clin. Cancer Res..

[33] Economidou F, Douka E, Tzanela M, Nanas S, Kotanidou A (2011). Thyroid function during critical illness. Hormones.

[34] Bischoff JR (1998). A homologue of Drosophila aurora kinase is oncogenic and amplified in human colorectal cancers. EMBO J..

[35] Tanaka E (2005). The clinical significance of Aurora-A/STK15/BTAK expression in human esophageal squamous cell carcinoma. Clin. Cancer Res..

[36] Xu J (2014). Aurora-A contributes to cisplatin resistance and lymphatic metastasis in non-small cell lung cancer and predicts poor prognosis. J. Transl. Med..

[37] Sen S (2002). Amplification/overexpression of a mitotic kinase gene in human bladder cancer. J. Natl Cancer Inst..

[38] Puig-Butille JA (2017). AURKA overexpression is driven by FOXM1 and MAPK/ERK activation in melanoma cells harboring BRAF or NRAS mutations: impact on melanoma prognosis and therapy. J. Invest. Dermatol..

[39] Li D (2003). Overexpression of oncogenic STK15/BTAK/Aurora A kinase in human pancreatic cancer. Clin. Cancer Res..

[40] Ochi T (2009). Aurora-A kinase: a novel target of cellular immunotherapy for leukemia. Blood.

[41] Wang J (2017). The Aurora-A-Twist1 axis promotes highly aggressive phenotypes in pancreatic carcinoma. J. Cell Sci..

[42] Tong T (2004). Overexpression of Aurora-A contributes to malignant development of human esophageal squamous cell carcinoma. Clin. Cancer Res..

[43] Reiter R (2006). Aurora kinase A messenger RNA overexpression is correlated with tumor progression and shortened survival in head and neck squamous cell carcinoma. Clin. Cancer Res..

[44] Neben K (2004). Microarray-based screening for molecular markers in medulloblastoma revealed STK15 as independent predictor for survival. Cancer Res..

[45] Landen CN (2007). Overexpression of the centrosomal protein Aurora-A kinase is associated with poor prognosis in epithelial ovarian cancer patients. Clin. Cancer Res..

[46] Chan EH, Santamaria A, Sillje HH, Nigg EA (2008). Plk1 regulates mitotic Aurora A function through betaTrCP-dependent degradation of hBora. Chromosoma.

[47] Liu Q (2004). Aurora-A abrogation of p53 DNA binding and transactivation activity by phosphorylation of serine 215. J. Biol. Chem..

[48] Anand S, Penrhyn-Lowe S, Venkitaraman AR (2003). AURORA-A amplification overrides the mitotic spindle assembly checkpoint, inducing resistance to Taxol. Cancer Cell.

[49] Lai CH (2017). Translational upregulation of Aurora-A by hnRNP Q1 contributes to cell proliferation and tumorigenesis in colorectal cancer. Cell Death Dis..

[50] Ice RJ (2013). NEDD9 depletion destabilizes Aurora A kinase and heightens the efficacy of Aurora A inhibitors: implications for treatment of metastatic solid tumors. Cancer Res..

[51] Huang YH, Wu CC, Chou CK, Huang CY (2011). A translational regulator, PUM2, promotes both protein stability and kinase activity of Aurora-A. PLoS ONE.

[52] Giubettini M (2011). Control of Aurora-A stability through interaction with TPX2. J. Cell Sci..

[53] Lee S, Cimica V, Ramachandra N, Zagzag D, Kalpana GV (2011). Aurora A is a repressed effector target of the chromatin remodeling protein INI1/hSNF5 required for rhabdoid tumor cell survival. Cancer Res..

[54] Yu Z (2017). SIX3, a tumor suppressor, inhibits astrocytoma tumorigenesis by transcriptional repression of AURKA/B. J. Hematol. Oncol..

[55] Nowak I (2021). MCPIP1 ribonuclease can bind and cleave AURKA mRNA in MYCN-amplified neuroblastoma cells. RNA Biol..

[56] Tong Y, Ben-Shlomo A, Zhou C, Wawrowsky K, Melmed S (2008). Pituitary tumor transforming gene 1 regulates Aurora kinase A activity. Oncogene.

[57] Sarkissian M, Mendez R, Richter JD (2004). Progesterone and insulin stimulation of CPEB-dependent polyadenylation is regulated by Aurora A and glycogen synthase kinase-3. Genes Dev..

[58] Katayama H, Zhou H, Li Q, Tatsuka M, Sen S (2001). Interaction and feedback regulation between STK15/BTAK/Aurora-A kinase and protein phosphatase 1 through mitotic cell division cycle. J. Biol. Chem..

[59] Taguchi S (2002). Degradation of human Aurora-A protein kinase is mediated by hCdh1. FEBS Lett..

[60] Kiat LS, Hui KM, Gopalan G (2002). Aurora-A kinase interacting protein (AIP), a novel negative regulator of human Aurora-A kinase. J. Biol. Chem..

[61] Jia L (2014). SMAD4 suppresses AURKA-induced metastatic phenotypes via degradation of AURKA in a TGFbeta-independent manner. Mol. Cancer Res..

[62] Irelan JT (2007). A role for IkappaB kinase 2 in bipolar spindle assembly. Proc. Natl Acad. Sci. USA.

[63] de Souza VB, Kawano DF (2020). Structural basis for the design of allosteric inhibitors of the Aurora kinase A enzyme in the cancer chemotherapy. Biochim. Biophys. Acta.

[64] Liewer S, Huddleston A (2018). Alisertib: a review of pharmacokinetics, efficacy and toxicity in patients with hematologic malignancies and solid tumors. Expert Opin. Investig. Drugs.

[65] Barr PM (2015). Phase II intergroup trial of alisertib in relapsed and refractory peripheral T-cell lymphoma and transformed mycosis fungoides: SWOG 1108. J. Clin. Oncol..

[66] O’Connor OA (2019). Randomized phase III study of alisertib or investigator’s choice (selected single agent) in patients with relapsed or refractory peripheral T-cell lymphoma. J. Clin. Oncol..

[67] Owonikoko TK (2020). Randomized phase II study of paclitaxel plus alisertib versus paclitaxel plus placebo as second-line therapy for SCLC: primary and correlative biomarker analyses. J. Thorac. Oncol..

[68] Falchook G (2019). Alisertib in combination with weekly paclitaxel in patients with advanced breast cancer or recurrent ovarian cancer: a randomized clinical trial. JAMA Oncol..

[69] Fletcher GC (2011). ENMD-2076 is an orally active kinase inhibitor with antiangiogenic and antiproliferative mechanisms of action. Mol. Cancer Ther..

[70] Diamond JR (2018). A phase II clinical trial of the Aurora and angiogenic kinase inhibitor ENMD-2076 for previously treated, advanced, or metastatic triple-negative breast cancer. Breast Cancer Res..

[71] Lheureux S (2018). A clinical and molecular phase II trial of oral ENMD-2076 in ovarian clear cell carcinoma (OCCC): a study of the princess margaret phase II Consortium. Clin. Cancer Res..

[72] Veitch Z (2019). A phase II study of ENMD-2076 in advanced soft tissue sarcoma (STS). Sci. Rep..

[73] Giles FJ (2013). MK-0457, an Aurora kinase and BCR-ABL inhibitor, is active in patients with BCR-ABL T315I leukemia. Leukemia.

[74] Seymour JF (2014). A phase 2 study of MK-0457 in patients with BCR-ABL T315I mutant chronic myelogenous leukemia and philadelphia chromosome-positive acute lymphoblastic leukemia. Blood Cancer J..

[75] Zhang P, Torres K, Liu X, Liu CG, Pollock RE (2016). An overview of chromatin-regulating proteins in cells. Curr. Protein Pept. Sci..

[76] Neigeborn L, Carlson M (1984). Genes affecting the regulation of SUC2 gene expression by glucose repression in Saccharomyces cerevisiae. Genetics.

[77] Wu JI, Lessard J, Crabtree GR (2009). Understanding the words of chromatin regulation. Cell.

[78] Wang X, Haswell JR, Roberts CW (2014). Molecular pathways: SWI/SNF (BAF) complexes are frequently mutated in cancer-mechanisms and potential therapeutic insights. Clin. Cancer Res..

[79] Clapier CR (2016). Regulation of DNA translocation efficiency within the chromatin remodeler RSC/Sth1 potentiates nucleosome sliding and ejection. Mol. Cell.

[80] Rowe CE, Narlikar GJ (2010). The ATP-dependent remodeler RSC transfers histone dimers and octamers through the rapid formation of an unstable encounter intermediate. Biochemistry.

[81] Kadoch C (2013). Proteomic and bioinformatic analysis of mammalian SWI/SNF complexes identifies extensive roles in human malignancy. Nat. Genet..

[82] Tagal V (2017). SMARCA4-inactivating mutations increase sensitivity to Aurora kinase A inhibitor VX-680 in non-small cell lung cancers. Nat. Commun..

[83] Wu C (2018). Targeting AURKA-CDC25C axis to induce synthetic lethality in ARID1A-deficient colorectal cancer cells. Nat. Commun..

[84] Versteege I (1998). Truncating mutations of hSNF5/INI1 in aggressive paediatric cancer. Nature.

[85] Biegel JA (2006). Molecular genetics of atypical teratoid/rhabdoid tumor. Neurosurg. Focus.

[86] Morozov A (2007). INI1 induces interferon signaling and spindle checkpoint in rhabdoid tumors. Clin. Cancer Res..

[87] Medina PP (2008). Frequent BRG1/SMARCA4-inactivating mutations in human lung cancer cell lines. Hum. Mutat..

[88] Jones DT (2012). Dissecting the genomic complexity underlying medulloblastoma. Nature.

[89] Shain AH (2012). Convergent structural alterations define SWItch/Sucrose NonFermentable (SWI/SNF) chromatin remodeler as a central tumor suppressive complex in pancreatic cancer. Proc. Natl Acad. Sci. USA.

[90] Wei XL (2014). Clinicopathologic and prognostic relevance of ARID1A protein loss in colorectal cancer. World J. Gastroenterol..

[91] Sun H (2011). E2f binding-deficient Rb1 protein suppresses prostate tumor progression in vivo. Proc. Natl Acad. Sci. USA.

[92] Minna JD, Roth JA, Gazdar AF (2002). Focus on lung cancer. Cancer Cell.

[93] Gong X (2019). Aurora A kinase inhibition is synthetic lethal with loss of the RB1 tumor suppressor gene. Cancer Discov..

[94] Lyu J (2020). Synthetic lethality of RB1 and aurora A is driven by stathmin-mediated disruption of microtubule dynamics. Nat. Commun..

[95] Alcaraz-Sanabria A (2017). Synthetic lethality interaction between aurora kinases and CHEK1 inhibitors in ovarian cancer. Mol. Cancer Ther..

[96] Lee JW (2019). Combined Aurora Kinase A (AURKA) and WEE1 inhibition demonstrates synergistic antitumor effect in squamous cell carcinoma of the head and neck. Clin. Cancer Res..

[97] Wang Y (2014). The negative interplay between Aurora A/B and BRCA1/2 controls cancer cell growth and tumorigenesis via distinct regulation of cell cycle progression, cytokinesis, and tetraploidy. Mol. Cancer.

[98] Do TV, Hirst J, Hyter S, Roby KF, Godwin AK (2017). Aurora A kinase regulates non-homologous end-joining and poly(ADP-ribose) polymerase function in ovarian carcinoma cells. Oncotarget.

[99] Kollareddy M (2012). Aurora kinase inhibitors: progress towards the clinic. Invest. N. Drugs.

[100] Zheng F (2016). Nuclear AURKA acquires kinase-independent transactivating function to enhance breast cancer stem cell phenotype. Nat. Commun..

